# Equitable psychiatry, telehealth, and the COVID-19 pandemic: Analysis of national data

**DOI:** 10.3389/fpubh.2023.1014302

**Published:** 2023-03-01

**Authors:** Thomas Yeatman, Joanne Enticott, Vinay Lakra, Graham Meadows

**Affiliations:** ^1^Department of Psychiatry, St Vincent's Hospital, Melbourne, VIC, Australia; ^2^Department of Psychiatry, Victorian Institute of Forensic Mental Health, Melbourne, VIC, Australia; ^3^Monash Centre for Health Research and Implementation, School of Public Health and Preventive Medicine, Monash University, Melbourne, VIC, Australia; ^4^Southern Synergy, School of Clinical Sciences at Monash Health, Monash University, Clayton, VIC, Australia; ^5^Mental Health Services, Northern Health, Melbourne, VIC, Australia; ^6^Department of Psychiatry, The University of Melbourne, Melbourne, VIC, Australia; ^7^Monash Centre for Health Research and Implementation, Monash University, Clayton, VIC, Australia; ^8^School of Primary and Allied Health Care, Monash University, Clayton, VIC, Australia; ^9^Centre for Mental Health, School of Population and Global Health, University of Melbourne, Melbourne, VIC, Australia; ^10^Monash Health, Dandenong, VIC, Australia

**Keywords:** psychiatry, telehealth, video consultations, equity, concentration index, telepsychiatry, COVID-19

## Abstract

**Background:**

At the beginning of the COVID-19 pandemic, it was foreseen that the number of face-to-face psychiatry consultations would suffer a reduction. In order to compensate, the Australian Government introduced new Medicare-subsidized telephone and video-linked consultations. This study investigates how these developments affected the pre-existing inequity of psychiatry service delivery in Australia.

**Methods:**

The study analyses five and a half years of national Medicare data listing all subsidized psychiatry consultation consumption aggregated to areas defined as Statistical Area level 3 (SA3s; which have population sizes of 30 k−300 k). Face-to-face, video-linked and telephone consultations are considered separately. The analysis consists of presenting rates of consumption, concentration graphs, and concentration indices to quantify inequity, using Socio Economic Indexes for Areas (SEIFA) scores to rank the SA3 areas according to socio-economic disadvantage.

**Results:**

There is a 22% drop in the rate of face-to-face psychiatry consultation consumption across Australia in the final study period compared with the last study period predating the COVID-19 pandemic. However, the loss is made up by the introduction of the new subsidized telephone and video-linked consultations. Referring to the same time periods, there is a reduction in the inequity of the distribution of face-to-face consultations, where the concentration index reduces from 0.166 to 0.129. The new subsidized video-linked consultations are distributed with severe inequity in the great majority of subpopulations studied. Australia-wide, video-linked consultations are also distributed with gross inequity, with a concentration index of 0.356 in the final study period. The effect of this upon overall inequity was to cancel out the reduction of inequity resulting from the reduction of face-to face appointments.

**Conclusion:**

Australian subsidized video-linked psychiatry consultations have been distributed with gross inequity and have been a significant exacerbator of the overall inequity of psychiatric service provision. Future policy decisions wishing to reduce this inequity should take care to reduce the risk posed by expanding telepsychiatry.

## Introduction

### Declining mental health in Australia, the impact of COVID-19 and expanding telehealth

Although there have been large increases in funding for mental health care, psychological distress levels, strongly associated with mental disorders, have been increasing in Australia over the last two decades (1, 2). Contributing to the failure to improve population mental health outcomes is an inequitable distribution of service provision (3). Socio-economic influences varying by location are important to consider here, and in Australia the Australian Bureau of Statistics calculates four Socio-economic Indexes for Areas (SEIFA) (4) each a different composite derived from 5-yearly Census data collections. These variables are intended to assist in the determination of areas that require funding and services, and to assist research into the relationship between socio-economic disadvantage and various health outcomes. Of the four SEIFA variables, the Index of Relative Socioeconomic Disadvantage (IRSD) has been most widely used in mental health epidemiology in Australia, and has been most regularly included in mental health related data releases. Populations that are socioeconomically more disadvantaged have a higher prevalence of mental illness and the most need, something established by numerous studies internationally since the 1930s and confirmed more recently in Australia with studies making use of the IRSD (as a measure of disadvantage) as included in national survey data sets (5–7). Yet in needing the most care, the most disadvantaged receive the least (8–10).

The inequity of provision has multiple contributing factors: one such is the common requirement of patients to make co-payments, often described as a “gap fee” above the Medicare rebated cost. The Better Access initiative was introduced in 2006 with the intention of increasing access to certain medical services, including psychiatry, by providing a proportionally large rebate of the scheduled cost of certain services (11). Legislation does not limit providers to charging only the scheduled fee, however. So, the co-payments may increase depending on the provider, and over a course of treatment co-payments may be substantial.

Another factor is the concentration of clinicians in less socioeconomically disadvantaged areas. In Australia, rural populations are often more socioeconomically deprived with additional positional disadvantage occasioned by large distances to travel to centers of care and limited travel options (12).

Upon this worsening state of affairs have unfolded the effects of the COVID-19 pandemic, affecting Australia since early 2020 (13). Telepsychiatry was identified as a vital tool in tackling the deficit of face-to-face consultations caused by the pandemic in various parts of the world (14, 15). Australia increased its Medicare-rebated telepsychiatry provision options from March 2020 (16), in an attempt to compensate for access problems for face-to-face consultations. However, the effect of COVID-19 and the corresponding increase in telehealth upon equitable access to psychiatry is not known. Given that inequitable access is thought to be a large factor behind declining mental health in Australia, it is important to understand how COVID-19 and increasing telepsychiatry have affected it, and what implications this holds for the future. The various sources of inequity in mental health care delivery will need to be addressed if improvements in Australian mental health are to be made, and telepsychiatry needs to be considered in this planning.

### Effect of COVID-19 upon face-to-face consultations

The COVID-19 pandemic prompted a variety of restrictive measures including curfews, international and local travel bans, face coverings, and others. Available data (17), indicates that Australia adopted similar measures to many developed countries. Although this data shows that some measures in Australia, such as the international travel ban, were adopted for longer than in some comparable countries. This reflects Australia's relatively more aggressive approach to COVID suppression, in part related to geographical factors such as its relative isolation. Within Australia, restrictive measures were enacted in different states for varying periods and to varying degrees, as is recorded in Australian government records (18, 19). Although travel was often permitted for medical consultations, there was a comparative shortage of clinicians (20), clinics reduced their capacities, and telepsychiatry consultations were considered as a less hazardous and more convenient alternative by both patients and doctors. The overall expected effect is a reduction in face-to-face consultations.

The same government records show that the restrictions were often enacted more severely in urban environments than in rural. So, it is expected that a greater reduction in face-to-face consultations would have occurred there.

On average, urban populations have greater social advantage than rural populations, and they receive more psychiatry consultations per person (9). So, the pandemic might have had a mitigating effect on this pre-existing inequitable uptake of face-to-face consultations. It is reasonable to expect that this effect has been largest in states where the difference in measures applied to rural and urban environments has been the greatest, and where the measures have been applied for the longest. According to the records mentioned, this difference was greatest in the State of Victoria, which we might expect to show a large reduction in inequity for face-to-face consultations, therefore.

### Effect of increased telepsychiatry upon equity

Telepsychiatry originally meant the delivery of mental health services *via* telecommunications devices, and included telephones, videoconferencing and other means of communication. In more recent use, it has increasingly meant video-conferencing only. For clarity, consultations will be referred to as either telephone (including mobile or landline) or video-linked in this discussion. Both modes of service delivery are used increasingly. It is thought that inequitable psychiatric service provision in Australia is partly due to clinicians living in more economically advantaged or urban areas (12). It has been hoped that telephone and video-linked consultations might overcome these geographical barriers to access. On the other hand, others have feared that video-linked consultations may present barriers to disadvantaged populations, which have less access to technology, are less familiar with its use, and have less private space available in which to receive confidential consultations (21). It is not known whether video-linked consultations create greater technological barriers than the geographical ones they remove. However, we are concerned that there may be an overall obstruction to access, because there is some evidence to suggest that the technological barriers are significant (22). By comparison, telephone consultations may surmount the barrier of distance equally well, but be less likely to erect a technological barrier. Again, greater uptake of these consultations is expected in states most subjected to restrictive measures, such as Victoria.

## Aims and hypotheses

In light of the discussion above, this study aims to explore the available data and test the hypotheses below.

1. During the pandemic, the inequity in the distribution of face-to-face consultations was reduced.
2. This effect was greatest in states where the difference in the application of restrictive measures between urban and rural environments was greatest, and applied for the longest.
3. The pandemic has been associated with an increase in telephone and video-linked consultations. This increase has been the greatest in urban environments and in states most subjected to restrictive measures.
4. Video-linked consultations are distributed with more inequity than either face-to-face appointments or telephone consultations.

## Materials and methods

### The data

Every psychiatry consultation receiving a Medicare subsidy in Australia is assigned an item code. Face-to-face psychiatry consultations are classified under Group A8 codes. On the 13th March 2020 subsidized telephone (Group A40 subgroup 6 codes) and video-linked (Group A40 subgroup 9 codes) psychiatry consultations were made widely available in response to the COVID-19 pandemic, and, at the time of writing, remain subsidized despite the recession of the pandemic.

The Royal Australian and New Zealand College of Psychiatrists (RANZCP) obtained enumerations of these consultation types by area from the Australian Government Department of Health. The request was made by one of the authors (VL) to inform ongoing work by the RANZCP with the Department of Health providing analyses to help guide future policy. The Medicare extract was in the form of the number of psychiatry consultations delivered to adults aged 18–64 years. This age range was provided because Australian health, employment and social care policies define the adult working age population aged 18–64 years. The data provided showed the number of consultations consumed within each Statistical Area 3 (SA3) area code, based upon the patient's residential address. There are 359 SA3s covering the whole of Australia without gaps or intersections (23). The Australian Bureau of Statistics has defined SA3s by clustering smaller areas (SA1 and SA2) such that the SA3 has a degree of internal homogeneity regarding socioeconomic disadvantage; SA3s also often have boundaries that are co-terminus with administrative boundaries, so these features along with the size of the overall data set of SA3s make them a good choice for examination of health variations. In contrast the next larger grouping, SA4 is designed with greater focus on labor markets. The residential population of each SA3 typically varies between 30,000 and 130,000 people.

In this study, data for each financial year since the 1st of July 2015 was provided (the Australian financial year ends 30th June). For the most recent available year at the time of the analysis, the provided data was truncated to the period 1st July 2020 until 30th November 2020.

This is a national data extract of routinely collected administrative data for all subsidized psychiatry consultations. Potential problems caused by the use of routinely collected health data are addressed elsewhere in this paper and the study results follow the RECORD reporting guidelines (24).

### Out of scope elements

Four elements were necessarily out of scope for this study, because the national data extract provided didn't include this information. These were:

1. Remote areas: Population numbers are low in a small number of remote Australian SA3s; therefore, for confidentially reasons the data custodian didn't make this data available.
2. Rural telehealth consultations in rural areas prior to March 2020: Prior to the pandemic, subsidized telehealth appointments were available to rural communities only, but a small number of subsidized telehealth consultations were consumed (25–27). Data describing the consumption of these appointments was not made available.
3. New and continuing patients: The national level data extract did not provide any information on whether consultations were accessed by new or continuing patients.
4. Gap fees (co-payments): The rebated video-linked and telephone psychiatry options that were introduced in March 2020 received the same rebate as the equivalent face-to-face items (28); however, the data extract did not provide any gap fee information, making any gap fee analysis out of scope.

### Elementary analyses

We examined Medicare subsidized psychiatry consultations for working age adults. We computed the number of consultations per 100,000 people per day in the time frames for which the data is available using the population figures from the Australian Bureau of Statistics in each SA3 for those aged 20–64 inclusive. The SA3s were combined to produce results for the whole of Australia, for each state, and for urban vs. rural populations. The SA3 areas do not always cover regions that could be considered entirely rural or entirely urban. Only an approximate classification of the SA3s into rural and urban was possible, therefore. This was achieved by classifying all those SA3s which lie inside the Greater Capital City Statistical Areas (which are defined along SA3 boundaries) as urban, and all those lying outside as rural. Restating this more simply, SA3s inside state capitals were classified as urban, and those outside as rural.

The Index for Relative Socio-economic Disadvantage (IRSD) for areas calculated by the Australian Bureau for Statistics from information in the 2016 census (SEIFA 2016) were used to rank SA3 areas by disadvantage. A fractional-rank IRSD score weighted by population was calculated for each SA3. The IRSD score was considered the most relevant index amongst the SEIFA for purposes of this analysis. It summarizes a combination of factors from residents in the area including income, education and type of work. A low score indicates high disadvantage.

In a small number of SA3s the service consumption was < 20 consultations per year. This occurred in locations of very low population density. In this circumstance the data was suppressed by Medicare to prevent the possibility of patients being identified; which is to say that no figure for the number of consultations was provided in these SA3s (element 1 of the Out of Scope section above). A sensitivity analysis was performed in which either: the suppressed SA3s were excluded from the analysis, or the number of consultations was assumed to be 0, or was assumed to be 20. All the approaches produced results that differed minimally, and had no bearing on the conclusions drawn (see Supplementary material). In the analyses reproduced here, the suppressed SA3s are assumed to have 0 consultations.

### Concentration graphs and indices

To measure the inequity of the distribution of consultations amongst SA3s, concentration graphs and concentration indices were computed by ranking SA3s by area disadvantage (using the IRSD scores).

Concentration graphs and concentration indices are a widely used method for measuring health inequity, and inequity of service delivery in the literature (29–31). Utilization of concentration indices can be seen in material produced by the World Bank (32) and the European Union Statistics on Income and Living Conditions (33).

A concentration graph is constructed by considering a ranking variable indicating social disadvantage. Data points are plotted using the individual's rank (scaled between 0 and 1) on the horizontal axis, and cumulative consumption of the health intervention (scaled between 0 and 1) on the vertical access. The graph is considered in relation to the diagonal between (0,0) and (1,1). A graph lying exactly on the diagonal represents a steady consumption of the health intervention where the rank conveys no advantage or disadvantage. However, a curve entirely below the line represents increased consumption by those of greater rank and decreased consumption by those of lesser rank. Whereas a curve entirely above the line represents the converse. A curve crossing the line may have more equivocal implications and requires more case specific consideration. The concentration index is obtained by computing twice the area between the diagonal and the concentration curve. By default, area above the diagonal is considered negative, and area below it positive. So, a positive value indicates a distribution favoring those of higher rank, and a negative value indicates a distribution favoring those of lower rank. By convention, an index of 0.2 indicates a high level of inequity (9). In this study, the statistics were computed for grouped data (by SA3), and the estimated concentration graphs and indices were plotted and computed using publicly available Stata modules (34, 35).

One reason that concentration graphs and indices are considered useful is that they provide a means of comparing inequity in different populations at different times. Nevertheless, their meaning requires consideration, especially when measuring concentration indices in populations that are examined separately and then combined; as occurs in this analysis—when states are analyzed separately and then as Australia whole. For example, two populations A and B may each have a concentration index of 0 with respect to a particular ranking variable, but the combined population of A and B together could have a concentration index that is negative, 0 or, positive. The outcome depends upon how the average rank and average consumption of A and B compare.

A further consideration must be made for populations where the need for an intervention is distributed heavily amongst those of lower rank; as it is in this case, where mental illness and the corresponding need for treatment is higher amongst people residing in areas with greater socioeconomic disadvantage—or lower IRSD (defining the rank in this case). A concentration index of 0 suggests equal service delivery amongst all social strata. However, the more disadvantaged need the service more. So, in this sense, the concentration index provides an underestimate of the inequity.

### Ethics approval

The Monash University Human Research Ethics Committee assessed the study proposal and granted an exemption because the non-identifiable data satisfied the criteria of the National Statement on Ethical Conduct in Human Research.

## Results

### Rate of consultation uptake in Australia as a whole

The number of consultations per 100,000 people of working age (20–64 years) per day in Australia in each financial year are shown in Table 1. The total rates of consumption of all consultation types are shown. In the financial year 2019–2020, the rate of consumption of video-linked and telephone consultations is calculated over the period for which the item numbers were available, i.e., from the 13th of March until the 30th of June-−110 days. However, the rate of consumption of face-to-face consultations and the total rate of consumption of all consultations is calculated over the period of 365 days (which is why the figures do not sum to the total rate of consumption during this financial year). In the final year 2020–2021, the rate of consumption is calculated over the period from July 1st 2020 until November 30th-−153 days—as the data is truncated at the end point. The same method of calculation applies to the other similar tables shown later.

**Table 1:** Consultations per 100,000 people of working age (20–64 years) per day—Australia.

**Financial year**	**Video-linked**	**Telephone**	**Face-to-face**	**Total**
2020–2021^†^	6.69^†^	9.33^†^	34.71^†^	50.73^†^
2019–2020	6.34^*^	9.77^*^	40.61	45.47
2018–2019	–	–	44.26	44.26
2017–2018	–	–	43.75	43.75
2016–2017	–	–	43.29	43.29
2015–2016	–	–	42.64	42.64

^*^Calculated over period 13/3/2020 to 30/6/2020.

^†^Calculated over period 1/7/2020 to 30/11/2020.

The results show a minimally increasing rate of face-to-face consultation uptake until the advent of COVID-19. The financial year 2019–2020 shows an 8.26% reduction in rate of face-to-face consultation uptake compared with 2018–2019, explained by the emergence of the COVID-19 pandemic at the end of the 2019–2020 financial year. However, the total rate of consultation uptake in 2019–2020 remains essentially unchanged because telephone and video-linked consultations compensated for the loss of face-to-face consultations. In the period July 1st to November 30th 2020, there is an even bigger reduction in the rate of uptake of face-to-face consultations compared with the year 2018–2019 (21.58%) because the entire period was affected by the pandemic. However, the total rate of consultation uptake increased 14.62% on 2018–2019 because Medicare supported telephone and video-linked consultations were available for the entire period. Thus, there was a bigger increase in the rate of consultation uptake over the final 2-year period, than over any prior 2-year period, due to the addition of Medicare funded telephone and video-linked consultations.

### Rate of consultation uptake by State

Consultation rates by states are shown in Table 2. Victoria, the second most populous state, had been subjected to much longer and more stringent lockdown conditions than any other state (19). The rate of face-to-face consultation uptake in the period 1st July to November 30th reflects this, with a 44.40% reduction compared with 2018–2019, the last financial year before the pandemic. The comparable figure for New South Wales, the most populous state, is 19.95%. A few of the less populous states, with less significant lockdowns, continued with similar rates of face-to-face consultation. Conversely, Victoria had a higher uptake of video and telephone consultations than seen in other states.

**Table 2:** Consultations per 100,000 people of working age (20–64 years) per day within each state.

	**Financial year**	**Video-linked**	**Telephone**	**Face-to-face**	**Total**
New South Wales	2020–2021^†^	6.28^†^	7.69^†^	34.04^†^	48.00^†^
	2019–2020	6.67^*^	8.29^*^	38.71	43.22
	2018–2019	–	–	42.52	42.52
	2017–2018	–	–	42.04	42.04
	2016–2017	–	–	40.67	40.67
	2015–2016	–	–	40.69	40.69
Victoria	2020–2021^†^	12.25^†^	16.44^†^	26.64^†^	55.33^†^
	2019–2020	7.73^*^	12.54^*^	42.70	48.80
	2018–2019	–	–	47.92	47.92
	2017–2018	–	–	47.97	47.97
	2016–2017	–	–	48.14	48.14
	2015–2016	–	–	47.53	47.53
Queensland	2020–2021^†^	4.65^†^	8.06^†^	46.21^†^	58.92^†^
	2019–2020	6.67^*^	11.50^*^	48.92	54.39
	2018–2019	–	–	51.81	51.81
	2017–2018	–	–	51.03	51.03
	2016–2017	–	–	50.71	50.71
	2015–2016	–	–	49.12	49.12
South Australia	2020–2021^†^	3.21^†^	7.17^†^	37.61^†^	48.00^†^
	2019–2020	5.81^*^	11.01^*^	37.84	42.91
	2018–2019	–	–	42.48	42.48
	2017–2018	–	–	42.79	42.79
	2016–2017	–	–	44.10	44.10
	2015–2016	–	–	43.86	43.86
Western Australia	2020–2021^†^	1.41^†^	2.44^†^	36.67^†^	40.52^†^
	2019–2020	2.85^*^	5.56^*^	32.45	34.98
	2018–2019	–	–	33.35	33.35
	2017–2018	–	–	30.94	30.94
	2016–2017	–	–	30.13	30.13
	2015–2016	–	–	29.13	29.13
Tasmania	2020–2021^†^	1.72^†^	3.77^†^	40.60^†^	46.10^†^
	2019–2020	2.22^*^	5.42^*^	38.42	40.73
	2018–2019	–	–	41.57	41.57
	2017–2018	–	–	45.06	45.06
	2016–2017	–	–	45.39	45.39
	2015–2016	–	–	43.89	43.89
Northern Territory	2020–2021^†^	1.18^†^	0.82^†^	8.29^†^	10.37^†^
	2019–2020	0.78^*^	1.14^*^	9.62	10.25
	2018–2019	–	–	9.53	9.53
	2017–2018	–	–	7.73	7.73
	2016–2017	–	–	8.54	8.54
	2015–2016	–	–	7.41	7.41
Australian Capital Territory	2020–2021^†^	4.61^†^	2.93^†^	25.90^†^	33.47^†^
	2019–2020	5.38^*^	2.96^*^	25.66	28.18
	2018–2019	–	–	25.57	25.57
	2017–2018	–	–	22.47	22.47
	2016–2017	–	–	20.13	20.13
	2015–2016	–	–	19.26	19.26

^*^Calculated over period 13/3/2020 to 30/6/2020.

^†^Calculated over period 1/7/2020 to 30/11/2020.

In every State, including Victoria, there was a higher increase in the total rate of consultation uptake in a 2-year period, despite the pandemic. Telepsychiatry provided the means to increase the rate, and in states more affected by the pandemic it more than compensated for the loss of face-to-face consultations.

### Rate of consultation uptake in rural vs. urban locations

Consultation rates by urban rural populations are shown in Tables 3A, B. The binary division led to a rural population of 4,340,820 and an urban population of 10,411,564. The sum is less than the population of Australia because the figures refer to 20–64 year-olds only.

**Table 3A:** Consultations per 100,000 people of working age (20–64 years) per day—Rural Australia.

**Financial year**	**Video-linked**	**Telephone**	**Face-to-face**	**Total**
2020–2021^†^	2.80^†^	5.88^†^	33.93^†^	42.62^†^
2019–2020	3.29^*^	6.55^*^	34.97	37.94
2018–2019	–	–	36.05	36.05
2017–2018	–	–	35.01	35.01
2016–2017	–	–	34.06	34.06
2015–2016	–	–	33.08	33.08

^*^Calculated over period 13/3/2020 to 30/6/2020.

^†^Calculated over period 1/7/2020 to 30/11/2020.

**Table 3B:** Consultations per 100,000 people of working age (20–64 years) per day—Urban Australia.

**Financial year**	**Video-linked**	**Telephone**	**Face-to-face**	**Total**
2020–2021^†^	8.32^†^	10.76^†^	35.03^†^	54.12^†^
2019–2020	7.62^*^	11.11^*^	42.96	48.60
2018–2019	–	–	47.69	47.69
2017–2018	–	–	47.40	47.40
2016–2017	–	–	47.13	47.13
2015–2016	–	–	46.62	46.62

^*^Calculated over period 13/3/2020 to 30/6/2020.

^†^Calculated over period 1/7/2020 to 30/11/2020.

The results show that, prior to the COVID-19 pandemic, the urban population attended face-to-face consultations at a significantly higher rate. The pandemic caused a much more precipitous fall in the rate of face-to-face consultation uptake compared with the rural population. It fell by 26.54% in the period 1st July 2020 to 30th November 2020 compared with the last pre-pandemic financial year 2018–2019. The corresponding fall in the rural population was 5.87%. The urban population is known to have undergone more severe restrictions during this period (18, 19). There was a much higher rate of non-face-to-face consultation uptake amongst the urban population, where the rate of telephone uptake was almost double that of the rural population and the rate of video-linked consultation uptake approximately triple. In the last financial year prior to the pandemic the urban population received 32.27% more consultations (of any type) than the rural population. In the period 1st July 2020 until 30th November 2020, they still received 26.97% more consultations despite the pandemic, due to the compensatory telehealth consultations.

### Concentration graphs and indices for Australia as a whole

**Figure 1:**
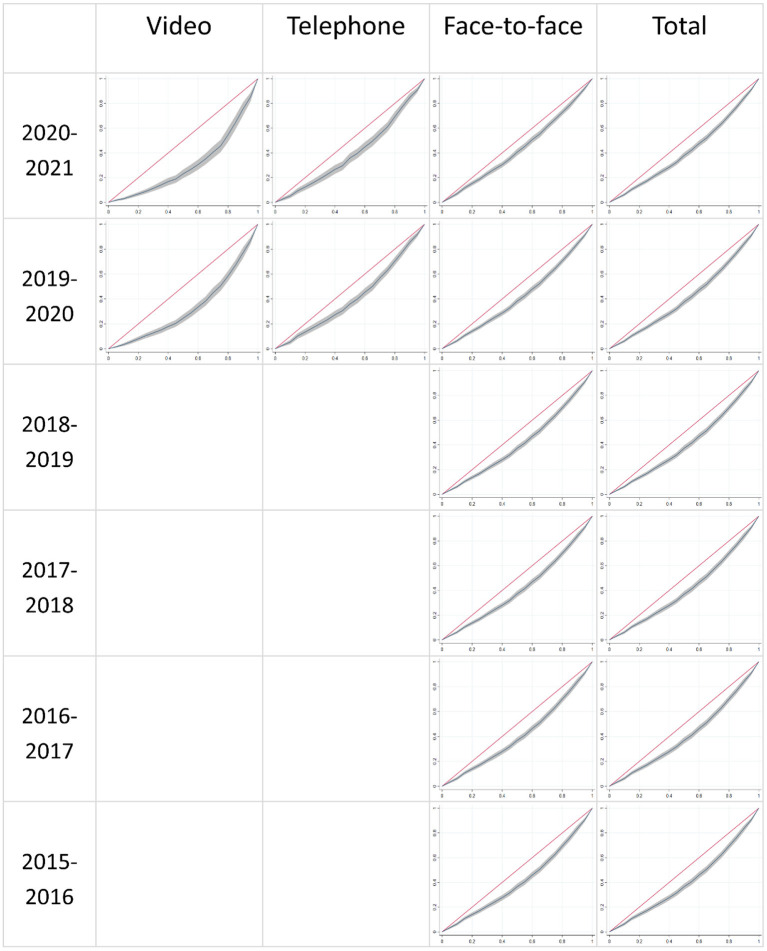
Concentration curves for Australia by consultation type and financial year. 2020–2021 financial year truncated to: July 1st 2020 to November 30th 2020.

Figure 1 shows the concentration graphs for the three consultation types—video-linked, telephone, and face-to face—for the 6 financial years, although the period for the last financial year is truncated to the 30th November 2020. It also shows the concentration graphs for total consultations for the 6 financial years. On each graph, the vertical axis shows the cumulative proportion of service consumed, and the horizontal axis shows the fractional rank of the SA3s (or cumulative proportion of the ranked population). The gray area around the line represents the 95% confidence interval. Table 4 shows the concentration indices corresponding to these graphs, with standard errors in brackets.

**Table 4:** Concentration indices for Australia with standard errors in brackets.

**Financial year**	**Video-linked**	**Telephone**	**Face-to-face**	**Total**
2020–2021^†^	0.356 (0.021)	0.155 (0.020)	0.129 (0.013)	0.163 (0.012)
2019–2020	0.326 (0.017)	0.140 (0.017)	0.160 (0.012)	0.166 (0.012)
2018–2019	–	–	0.166 (0.012)	0.166 (0.012)
2017–2018	–	–	0.166 (0.013)	0.166 (0.013)
2016–2017	–	–	0.166 (0.013)	0.166 (0.013)
2015–2016	–	–	0.171 (0.013)	0.171 (0.013)

^†^2020–2021 financial year truncated to: July 1st 2020 to November 30th 2020.

The results show that there is a stable inequity in the distribution of face-to-face consultations in the years prior to the pandemic with a concentration index of ~0.166. This figure drops during the year the pandemic began, and then drops further to 0.129 in the most recent period 1st July 2020 to 30th November 2020. This corresponds to a larger reduction in face-to-face consultations in urban (and overall more privileged) areas, compared with regional and remote areas, where there were longer and more stringent lockdowns.

On the other hand, there is an introduction of non-face-to-face consultations that is inequitable. In the most recent period, telephone consultations are distributed with a concentration index of 0.155, not dissimilar to the inequity prior to the pandemic; but video-linked consultations are distributed with a concentration index of 0.356, indicating very large inequity.

### Concentration indices by State

Concentration indices for each state are shown in Table 5. For face-to-face consultations, the three most populous states (New South Wales, Victoria, and Queensland) have concentration indices that are often significantly higher than what they are for the combined Australian face-to-face concentration indices shown in Table 4. This illustrates that the concentration index for a combined population is not easily deduced from the concentration indices of the subpopulations. It is explained by various interstate discrepancies, such as: Queensland having a higher average rate of consultation (Table 2), but a greater average level of social disadvantage (measured by the population-weighted IRSD score), compared with Victoria and New South Wales.

**Table 5:** Concentration indices for each state with standard errors in brackets.

	**Financial year**	**Video-linked**	**Telephone**	**Face-to-face**	**Total**
New South Wales	2020–2021^†^	0.400 (0.029)	0.160 (0.026)	0.164 (0.021)	0.194 (0.021)
	2019–2020	0.397 (0.029)	0.140 (0.029)	0.179 (0.021)	0.187 (0.022)
	2018–2019	–	–	0.182 (0.022)	0.182 (0.022)
	2017–2018	–	–	0.182 (0.023)	0.182 (0.023)
	2016–2017	–	–	0.182 (0.023)	0.182 (0.023)
	2015–2016	–	–	0.188 (0.023)	0.188 (0.023)
Victoria	2020–2021^†^	0.337 (0.025)	0.136 (0.020)	0.157 (0.023)	0.191 (0.018)
	2019–2020	0.322 (0.028)	0.140 (0.021)	0.199 (0.019)	0.200 (0.019)
	2018–2019	–	–	0.204 (0.019)	0.204 (0.019)
	2017–2018	–	–	0.202 (0.019)	0.202 (0.019)
	2016–2017	–	–	0.202 (0.019)	0.202 (0.019)
	2015–2016	–	–	0.209 (0.020)	0.209 (0.020)
Queensland	2020–2021^†^	0.291 (0.035)	0.185 (0.037)	0.111 (0.021)	0.136 (0.021)
	2019–2020	0.282 (0.031)	0.196 (0.035)	0.142 (0.021)	0.150 (0.021)
	2018–2019	–	–	0.159 (0.021)	0.159 (0.021)
	2017–2018	–	–	0.166 (0.022)	0.166 (0.022)
	2016–2017	–	–	0.166 (0.022)	0.166 (0.022)
	2015–2016	–	–	0.169 (0.022)	0.169 (0.022)
South Australia	2020–2021^†^	0.266 (0.053)	0.129 (0.030)	0.194 (0.030)	0.189 (0.030)
	2019–2020	0.271 (0.040)	0.162 (0.041)	0.187 (0.030)	0.189 (0.031)
	2018–2019	–	–	0.185 (0.029)	0.185 (0.029)
	2017–2018	–	–	0.199 (0.031)	0.199 (0.031)
	2016–2017	–	–	0.213 (0.033)	0.213 (0.033)
	2015–2016	–	–	0.207 (0.033)	0.207 (0.033)
Western Australia	2020–2021^†^	0.230 (0.42)	0.118 (0.48)	0.172 (0.34)	0.170 (0.34)
	2019–2020	0.248 (0.50)	0.132 (0.40)	0.180 (0.33)	0.179 (0.33)
	2018–2019	–	–	0.189 (0.33)	0.189 (0.33)
	2017–2018	–	–	0.183 (0.33)	0.183 (0.33)
	2016–2017	–	–	0.184 (0.32)	0.184 (0.32)
	2015–2016	–	–	0.198 (0.31)	0.198 (0.31)
Tasmania	2020–2021^†^	0.240 (0.68)	0.238 (0.71)	0.099 (0.41)	0.115 (0.44)
	2019–2020	0.257 (0.63)	0.153 (0.82)	0.102 (0.53)	0.107 (0.54)
	2018–2019	–	–	0.096 (0.57)	0.096 (0.57)
	2017–2018	–	–	0.105 (0.54)	0.105 (0.54)
	2016–2017	–	–	0.112 (0.65)	0.112 (0.65)
	2015–2016	–	–	0.100 (0.59)	0.100 (0.59)
Northern Territory	2020–2021^†^	−0.002 (0.165)	0.052 (0.186)	0.031 (0.125)	0.021 (0.126)
	2019–2020	−0.099 (0.190)	−0.100 (0.252)	−0.018 (0.110)	−0.023 (0.112)
	2018–2019	–	–	−0.035 (0.131)	−0.035 (0.131)
	2017–2018	–	–	−0.033 (0.096)	−0.033 (0.096)
	2016–2017	–	–	−0.053 (0.109)	−0.053 (0.109)
	2015–2016	–	–	−0.086 (0.163)	−0.086 (0.163)
Australian Capital Territory	2020–2021^†^	0.053 (0.065)	0.028 (0.064)	0.009 (0.063)	0.016 (0.062)
	2019–2020	−0.006 (0.049)	0.047 (0.076)	0.003 (0.064)	0.004 (0.063)
	2018–2019	–	–	0.014 (0.063)	0.014 (0.063)
	2017–2018	–	–	0.033 (0.071)	0.033 (0.071)
	2016–2017	–	–	0.045 (0.081)	0.045 (0.081)
	2015–2016	–	–	0.046 (0.088)	0.046 (0.088)

^†^2020–2021 financial year truncated to: July 1st 2020 to November 30th 2020.

Prior to the pandemic, the concentration indices for face-to-face consultations were essentially stable. However, for the three most populous states there is a reduction in the concentration index in the time periods affected by the pandemic, namely the 2019–2020 financial year and the period 1st July 2020 until 30th November 2020. This was pronounced in Victoria where Greater Melbourne suffered much longer lockdowns than the rest of Australia, so consultations for more urban and socially advantaged populations were disproportionately reduced, restoring some equity to the distribution of face-to-face consultations. Queensland also showed a marked reduction in the concentration index for face-to-face consultations.

The remaining five, less populous, states did not suffer severe restrictions during the study periods (18, 19). Correspondingly, the concentration indices for face-to-face consultations in these states show less change (Table 5).

Amongst the states with a population of greater than 1 million, video-linked consultations were distributed highly inequitably. In the period 1st July 2020 to 30th November 2020, NSW had a concentration index of 0.400. Victoria, the biggest consumer of video-linked consultations, had a concentration index of 0.337 in this period. This was not the case with telephone linked consultations in these larger states, where the concentration index for telephone consultations in the period 1st July 2020 to 30th November 2020 was less than that for face-to-face consultations, excepting Queensland.

### Concentration indices for urban vs. rural populations

In comparing urban or rural populations, data for individual states has been selected. Selecting the entirety of the rural or urban population leads to a degree of confounding because the progression of the concentration indices over time are affected by changing levels of restrictions within the states or cities, while there are also different levels of overall social advantage within each state. This can cause a confusing interaction. The data for Victoria has been selected for examination below, because social restrictions applied for a much larger portion of the final time period studied, and the social restrictions were applied with greater uniformity across the capital or rural regions than they were in other states undergoing significant restriction, such as NSW.

The Victorian population (20–64 year-olds) divides into 806,909 living rurally and 3,156,978 in urban Victoria (Greater Melbourne). Tables 6A, B show the concentration indices for these populations.

**Table 6A:** Concentration indices for urban Victoria (Greater Melbourne).

**Financial year**	**Video-linked**	**Telephone**	**Face-to-face**	**Total**
2020–2021^†^	0.309 (0.029)	0.110 (0.017)	0.208 (0.021)	0.202 (0.019)
2019–2020	0.313 (0.032)	0.118 (0.018)	0.216 (0.020)	0.213 (0.020)
2018–2019	–	–	0.211 (0.020)	0.211 (0.020)
2017–2018	–	–	0.205 (0.019)	0.205 (0.019)
2016–2017	–	–	0.201 (0.019)	0.201 (0.019)
2015–2016	–	–	0.208 (0.021)	0.208 (0.021)

^†^2020–2021 financial year truncated to: July 1st 2020 to November 30th 2020.

**Table 6B:** Concentration indices for rural Victoria.

**Financial year**	**Video-linked**	**Telephone**	**Face-to-face**	**Total**
2020–2021^†^	0.130 (0.045)	0.120 (0.044)	0.075 (0.022)	0.093 (0.024)
2019–2020	0.152 (0.059)	0.142 (0.058)	0.077 (0.025)	0.084 (0.025)
2018–2019	–	–	0.088 (0.026)	0.088 (0.026)
2017–2018	–	–	0.078 (0.028)	0.078 (0.028)
2016–2017	–	–	0.086 (0.027)	0.086 (0.027)
2015–2016	–	–	0.091 (0.025)	0.091 (0.025)

^†^2020–2021 financial year truncated to: July 1st 2020 to November 30th 2020.

The results show that within urban Victoria (Greater Melbourne) the concentration index for face-to-face consultations remains essentially stable. So, even though the pandemic reduced the number of consultations, it did so to the same degree across social strata. This is unsurprising as Greater Melbourne underwent essentially uniformly stringent restrictions. The results, supplied in the Supplementary material, for urban NSW and urban Queensland, the other two most populous states, are similar in this respect. When Victorian non-face-to-face consultations are additionally considered, the results show that video-linked consultations exacerbate the existing inequity, but that telephone consultations mitigate it. Such that, there is only a small change in the concentration index for the total number of consultations compared with previous years. The results for urban NSW and urban Queensland are similar in these respects also.

There is a small decrease in the concentration index for face-to-face consultations in rural Victoria compared with pre-pandemic levels. When compared with the more precipitous drop in the concentration index for face-to-face consultations for the whole of Victoria (displayed in Table 5), this suggests that the large state-wide decrease is caused by the differential effect of the pandemic upon rural and urban Victoria. Face-to face consultations were relatively preserved in rural communities which are more disadvantaged than urban ones, resulting in a more equitable distribution of face-to-face consultations for the combined population.

Video-linked consultations are a significant exacerbator of inequity within both the urban and rural populations in Victoria. This is true for all states in the most recent period, with the exception of the Norther Territory where the standard error is too high to allow useful interpretation anyway. Conversely, telephone consultations are a mitigator of inequity in both urban and rural Victorian populations. This result is not consistent across states, and exceptions include rural Queensland where telephone consultations have exacerbated inequity.

## Discussion

### Key findings considered in relation to study hypotheses

#### The pandemic reduced the inequitable distribution of face-to-face consultations

Examination of almost 6 years of population-level, Australia-wide psychiatry service data show that face-to-face psychiatry consultations have been inequitably distributed in favor of SA3s with less social disadvantage. However, the COVID-19 pandemic partially mitigated this inequity. This is clearly shown in concentration indices for Australia taken as a whole.

#### Greater reduction of inequity for face-to-face consultations where there were greater urban restrictions

In focusing upon the data for Victoria (experienced most restrictions), there is a large reduction in the inequity of face-to-face consultations uptake, which is not apparent when considering the rural-Victorian and urban-Victorian data separately. This suggests that the state-wide reduction in inequity was likely caused primarily by the differential between the restrictions applied across the urban-rural divide. The same is apparent in the data for NSW, Furthermore, the reduction in the concentration index for face-to-face consultations is most precipitous in Victoria (Supplementary material). These findings also support the second hypothesis.

#### Uneven distribution of increasing telepsychiatry consultations favoring urban environments and more heavily restricted states

The data show that video-linked consultations were higher in the most socially restricted state, Victoria, and that consumption continued to increase into the final study period. Whereas in states less subjected to restrictions, there was a less avid uptake of telepsychiatry. Furthermore, in the final period studied, when initial COVID-19 restrictions were relaxed in the majority of states other than Victoria, there is a slight decrease in the telepsychiatry usage. Also, telepsychiatry consultations per capita were higher in urban SA3s compared with rural ones. So, the third hypothesis is supported.

#### Greater inequity in video-linked consultations

Examining video-linked consultations first, it is plain that the concentration indices are very high Australia-wide and much greater than those typically applying to face-to-face items. Telephone items are distributed with inequity more typical of face-to face items. The final hypothesis is supported.

It is worth pausing to consider the strength of the findings for video-linked consultations. They show that the observed inequity is not merely an artifact of the pandemic. It could be argued that video-linked consultations were distributed with severe inequity primarily because they have taken up by the, on average, more privileged urban communities, who were *obliged* to do so having missed out on the face-to-face consultations that they enjoyed pre-pandemic. Or put differently, if there had been no pandemic, the urban communities would not have experienced the relative lack of consultations that drove them to consume video-linked ones, and consequently no great inequity of video-linked consultations would have been observed. However, this is not borne out by the findings. The concentration indices for urban Victoria considered alone show that all social strata missed out equally on face-to-face consultations (because there is minimal change in the concentration index for this population). So, all social strata were equally driven to consume telehealth consultations, but these were nevertheless distributed highly inequitably amongst this population. Video-linked consultations appear to be an exacerbator of inequity when COVID-19 restrictions are kept equal across social strata. The same is observed in the results for rural Victoria considered separately. Indeed, in virtually all the subpopulations studied, the concentration indices for video-linked consultations were conspicuously high.

Similarly, it could be argued that the Medicare-rebated video-linked and telephone items available to rural locations pre-pandemic have distorted the analysis (even though uptake was low). The data regarding their consumption was not provided to us, but if rural areas were already receiving some rebated telepsychiatry, then it might be argued that the observed inequity in telepsychiatry uptake is explained by the fact that urban areas (on average less disadvantaged) were now catching up in their telepsychiatry use, now that a rebate was made available to all. However, this argument does not bear weight, again because of the very high concentration indices for telepsychiatry observed within urban Victoria and rural Victoria, considered separately (likewise observed when the rural and urban populations are considered separately in the other states).

The striking conclusion is that video-linked consultations present significant barriers to people living in socio-economically disadvantaged areas. This is a significant and robust finding because it has been deduced by quantifying the inequity found in a large national data set, something that other studies in this area have not done.

### Comparison with existing literature

Other studies report a rapid expansion in Australian telepsychiatry at the onset of the pandemic (27), and some studies have found that there was a greater uptake of video-linked telehealth consultations amongst less disadvantaged populations during the COVID-19 pandemic (22). They have also shown that, amongst patients who attend non-face-to-face consultations, disadvantaged patients are more likely to attend *via* telephone than advantaged patients. This could be a problem as there is some evidence to suggest video-linked appointments are preferable for patient satisfaction and diagnostic accuracy (21, 36).

Another study raised inequitable access as a potential problem arising from the introduction of telepsychiatry during the COVID-19 pandemic. It recommended that services record and track telepsychiatry usage to monitor the effects of the “digital chasm” between classes, which the paper referred to as an increasingly important social determinant of health (37).

It is unclear whether mental health problems increased or not during the COVID-19 pandemic. One Australian study showed an increased demand for services but indicated that mental health had not declined (38). However, various studies give reason to believe that the COVID-19 pandemic may have increased the prevalence of mental health problems disproportionately for the disadvantaged (39–41). They cite various intersecting problems combining with the pandemic to result in a greater vulnerability to mental health problems, including: poverty, poor housing, reduced access to education and employment, worse physical health outcomes, and discrimination, to name a few. If the burden of need has increased amongst the disadvantaged, this would not be reflected in the concentration indices of service delivery, and is not part of the analysis conducted in this paper.

An extensive and enlightening evaluation of the aforementioned Better Access initiative has recently been conducted, which comprises a number of subsidiary papers (11). Although drawing a number of positive conclusions, it highlights a number of concerns regarding worsening inequitable consumption of the treatments supported by the initiative. Their evaluation is broadly encouraging of telepsychiatry, stating that it increased access during the pandemic. However, our findings indicate that this was not an equitable increase. They also state existing patients are more likely to receive telepsychiatry than new patients for various reasons. Our study does not provide further information on this point, but considerations around this are evidently important for future service planning.

### Implications of the findings

Here we have seen a rare example in of a decrease in a measure of inequity, where face-to-face consults declined in urban areas, which are less disadvantaged on average. However this did not involve any increase to those with greater need, so was not necessessarily a welcome change.

The findings draw attention to the effect of COVID-19 restrictions on access to mental health care. The benefits of containing viral transmission are seen as coming with signitficant adverse effects as has been seen in other areas of healthcare.

We see here very striking inequities in video-linked consultations. Concentration indices of typically above 0.2 and up to 0.4 are indicative of massive disparities. The highest of these levels is unprecedented in descriptions of Medicare funding inequities in Australia (9). Video-linked consultations have the potential to reach underserved people who have problems with travel, including people in poorer outer city suburbs, but this does not seem to have occurred. Rather, the increased service volume has gone to people more likely to have good internet access and the hardware and privacy necessary to access this modality. While many of these recipients are likely to have had perfectly valid reasons for accessing Better Access and other Medicare-subsidized services, the high volume of video linked services they have received has come at the cost of poor delivery to those in areas with higher need. It is probable that unmet need increased amongst some disadvantaged urban populations because they missed out, not only on face-to-face consultations due to social restrictions, but also on the compensatory video consultations due to inequitable delivery. Without major structural changes, encouragement of video-linked delivery seems to be a strategy likely to increase inequity rather than decrease it.

Some of the explanations for the inaccessibility of telehealth to socioeconomically disadvantaged populations are explicitly described by the variables contributing to the IRSD index: low income, making it difficult to obtain adequate technology; overcrowded living conditions, hampering privacy; lack of internet connection; and poor English, impairing access and communication (4). However, it is likely that many other factors associated with socio-economic disadvantage conspire to further reduce access. The Andersen model is a widely used theoretical framework that explores how these factors result in inequitable health service distribution. A recent paper describing the model identified that there is a paucity of research applying the Andersen model to technological innovations such as telehealth (42). This suggests a fruitful direction for future research. In addition to advancing such theory-based explorations, future research could examine the trend over time as the post-COVID-19 pandemic circumstances become clearer. It could also examine other disciplines including psychology.

### Limitations

The truncation of the data in the last financial year is a possible source of bias. It is likely that some seasonal variation occurs in consultation consumption and that the results will be affected because only part of the year is represented. However other studies indicate that this effect is not large (27), and the large effects caused by COVID and the increase in telehealth significantly outweigh any bias of this kind.

Some other studies have reported on item number consumption on a quarterly basis (27, 43, 44). Ideally, finer grained time linked data of this kind would have been helpful, as it could have been linked to changes in COVID-19 restrictions as they varied in the different states. It would not have been possible to obtain such data linked to SA3, however, because lower numbers of consultations in shorter time frames would have resulted in greater suppression of data to preserve patient anonymity. It was in one sense serendipitous that the final time period was truncated to the 30th November 2020, because this coincided with a significant period of Victorian COVID-19 spread and associated social restriction. Similarly, data linked to finer grained geographical locations, such as SA1s, might have been preferable, and would have allowed a less crude classification into urban and rural. However, it is again likely that this would have mandated more data suppression to preserve anonymity.

It is possible that the results are subject to a degree of confounding, caused, for example, by differing conditions in each state. This particular problem has been alleviated where possible, by considering each state separately in the analysis. The most perturbing confounder is likely to be the preference of young adults to live in urban environments. This is likely to impact most upon the results for video-linked consultations, where younger people are more comfortable with the technology required to facilitate these. However, the high concentration indices for telehealth consultations within both urban and rural environments in each state (Table 6, Supplementary Tables) show that this is not a large source confounding, and would not upset any of the conclusions drawn. There are arguments for and against restricting the data to the working age population. The data provided to us left us no choice in this regard, but it might be reconsidered in future analyses.

A small number of SA3s could not be included in the analysis due to incomplete data. However, their combined population was a very small percentage of the total populations and is very unlikely to have affected the results significantly. The results most affected by this problem are likely to have been those for Western Australia. The locations had very low IRSD scores and very low populations densities, and it is likely that if they could have been included, the reported inequities would have been even more pronounced than those shown.

### Rethinking funding models

The dramatically inequitable expansion of largely public-funded services observed in this paper, occurring at a time of unprecedented community need, has arisen in the context of a market-driven service model (3). This change seems to be a further example (9) of the failure of this model to deliver on Medicare's promise of universality. However, the current model is not the only possible way of funding Australian healthcare. Other funding models from around the world that could be adapted for Australian settings include capitation funding models (45). In Australia, proposals for an alternative known as “Medicare Select” (46) were stalled for political reasons over a decade ago. This could have introduced a new kind of wider competition to the health insurance market. It was a model that could have stimulated service models with a greater guarantee of actually delivering services to people as insurance purchasers rather than creating notional entitlements that are not actually in any kind of reach for those in need. Offered a range of insurance products, people with less financial resources might opt for insurance coverage that was optimized for access to service and low co-payments over the current Medicare model, which seeks to allow choice even where there may be considerable co-payments. More recently, in Australia and elsewhere, there is talk of enrolled-patient populations as a way to make funding more population needs based (3). We will not go so far herein as to suggest a specific solution to tackle inequitable metal health service provision in Australia, as any solution will be complex. Instead, these study findings provide a real-world population-level case study showing that it is not always obvious how relatively minor changes to an existing system can actually be counterproductive to improving access in a universal care setting. Some more radical remodeling is likely to be required, as complex adaptive systems facilitating healthcare access do not necessarily achieve the outcome that was intended.

## Conclusion

This study suggests that video-linked psychiatry can create greater technological barriers for the socially disadvantaged than the geographical barriers it overcomes. The combined effect of telephone and video-linked consultations was to exacerbate inequity, such that the reduction in inequity caused by the loss of urban face-to-face consultations, was essentially canceled out.

As the pandemic recedes, face-to-face consultations will rebound in urban areas and the prior inequity in face-to-face consultations is likely to be restored. However, video-linked psychiatry will not disappear (47, 48). The pandemic has catapulted urban populations further into the new era of telepsychiatry, than it has rural populations. Urban mental health services are now better prepared to deliver video-linked consultations, and urban populations are better prepared to participate in them. Yet the hope for telepsychiatry was that its greatest use would be in overcoming the distances that separate disadvantaged rural patients from the services they more desperately need.

This is not the only concern. The technology required to access video-linked consultations presents significant barriers to the disadvantaged in general; those in urban locations as well as rural. Whether the problems in accessing video-linked consultations relate to poorly resourced patients, or services, or both, they need to be tackled. Policy makers have been attracted to video-linked psychiatry because there is evidence to suggest that it produces similar outcomes for decreased expenditure (21). However, the introduction of the item number for video-linked consultations without additional support for services and patients has exacerbated inequity. This may soon be further exacerbated by a recent policy announcement to reduce Medicare supported telephone items but extend video-linked ones. This is concerning, not least because inequitable service provision is likely to be have been a key factor undermining mental health outcomes in Australia (2).

## Data availability statement

The data analysed in this study can be obtained after approval from Services Australia, who can provide researchers access to the same Medicare data analysed in this study.

## Ethics statement

The Monash University Human Research Ethics Committed assessed the study proposal and granted an exemption because the non-identifiable data satisfied the criteria of the National Statement on Ethical Conduct in Human Research. Written informed consent for participation was not required for this study in accordance with the national legislation and the institutional requirements.

## Author contributions

VL liaised with the Australian Department of Health to obtain the data. JE was the senior statistician who oversaw the analysis. GM provided advice on the analysis of the data. TY performed the analysis and wrote the first draft of the paper. All authors provided critical inputs. All authors contributed to the article and approved the submitted version.
