# Supplementary material for: Equitable psychiatry, telehealth, and the COVID-19 pandemic: Analysis of national data
Source: Front Public Health. 2023 Mar 1;11:1014302. doi: 10.3389/fpubh.2023.1014302 (PMC10014820; doi:10.3389/fpubh.2023.1014302)
Supplement: Supplementary file 1 [file Table_1.DOCX]

# Supplementary Tables

## More concentration indices

Table S1 Concentration indices for urban NSW (Greater Sydney) with standard errors in brackets

| Financial Year | Video-linked | Telephone | Face-to-face | Total |
| --- | --- | --- | --- | --- |
| 2020-2021† | 0.346 (0.047) | 0.158 (0.027) | 0.217 (0.022) | 0.227 (0.025) |
| 2019-2020 | 0.344 (0.047) | 0.143 (0.024) | 0.213 (0.025) | 0.216 (0.026) |
| 2018-2019 | - | - | 0.206 (0.026) | 0.206 (0.026) |
| 2017-2018 | - | - | 0.204 (0.026) | 0.204 (0.026) |
| 2016-2017 | - | - | 0.203 (0.027) | 0.203 (0.027) |
| 2015-2016 | - | - | 0.206 (0.026) | 0.206 (0.026) |

† 2020-2021 financial year truncated to: July 1^st^ 2020 to November 30^th^ 2020

Table S2 Concentration indices for rural NSW with standard errors in brackets

| Financial Year | Video-linked | Telephone | Face-to-face | Total |
| --- | --- | --- | --- | --- |
| 2020-2021† | 0.208 (0.057) | 0.098 (0.061) | 0.072 (0.031) | 0.085 (0.032) |
| 2019-2020 | 0.256 (0.077) | 0.054 (0.070) | 0.078 (0.031) | 0.081 (0.032) |
| 2018-2019 | - | - | 0.082 (0.032) | 0.082 (0.032) |
| 2017-2018 | - | - | 0.086 (0.035) | 0.086 (0.035) |
| 2016-2017 | - | - | 0.069 (0.033) | 0.069 (0.033) |
| 2015-2016 | - | - | 0.066 (0.035) | 0.066 (0.035) |

† 2020-2021 financial year truncated to: July 1^st^ 2020 to November 30^th^ 2020

Table S3 Concentration indices for urban Queensland (Greater Brisbane) with standard errors in brackets

| Financial Year | Video-linked | Telephone | Face-to-face | Total |
| --- | --- | --- | --- | --- |
| 2020-2021† | 0.203 (0.028) | 0.078 (0.028) | 0.136 (0.018) | 0.133 (0.018) |
| 2019-2020 | 0.209 (0.026) | 0.094 (0.027) | 0.141 (0.019) | 0.141 (0.019) |
| 2018-2019 | - | - | 0.143 (0.020) | 0.143 (0.020) |
| 2017-2018 | - | - | 0.141 (0.020) | 0.141 (0.020) |
| 2016-2017 | - | - | 0.139 (0.019) | 0.139 (0.019) |
| 2015-2016 | - | - | 0.147 (0.020) | 0.147 (0.020) |

† 2020-2021 financial year truncated to: July 1^st^ 2020 to November 30^th^ 2020

Table S4 Concentration indices for rural Queensland with standard errors in brackets

| Financial Year | Video-linked | Telephone | Face-to-face | Total |
| --- | --- | --- | --- | --- |
| 2020-2021† | 0.207 (0.035) | 0.224 (0.045) | 0.060 (0.033) | 0.081 (0.032) |
| 2019-2020 | 0.221 (0.047) | 0.239 (0.047) | 0.095 (0.033) | 0.104 (0.033) |
| 2018-2019 | - | - | 0.122 (0.030) | 0.122 (0.030) |
| 2017-2018 | - | - | 0.131 (0.031) | 0.131 (0.031) |
| 2016-2017 | - | - | 0.134 (0.030) | 0.134 (0.030) |
| 2015-2016 | - | - | 0.129 (0.030) | 0.129 (0.030) |

† 2020-2021 financial year truncated to: July 1^st^ 2020 to November 30^th^ 2020

## Sensitivity analysis

Table S5 Concentration indices for Australia – discarding the SA3s with supressed data prior to computation

| Financial Year | Video-linked | Telephone | Face-to-face | Total |
| --- | --- | --- | --- | --- |
| 2020-2021† | 0.35361996† | 0.15311661† | 0.12886598† | 0.16345855† |
| 2019-2020 | 0.32222078 | 0.13902668 | 0.15995341 | 0.16561478 |
| 2018-2019 | - | - | 0.16632954 | 0.16632954 |
| 2017-2018 | - | - | 0.16607318 | 0.16607318 |
| 2016-2017 | - | - | 0.16604542 | 0.16604542 |
| 2015-2016 | - | - | 0.17106250 | 0.17106250 |

†Calculated over period 1/7/2020 to 30/11/2020

Table S6 Concentration indices for Australia – entering 0 consultations for SA3s where the data has been suppressed.

| Financial Year | Video-linked | Telephone | Face-to-face | Total |
| --- | --- | --- | --- | --- |
| 2020-2021† | 0.35553981† | 0.1545492† | 0.12886598† | 0.16345855† |
| 2019-2020 | 0.32600785 | 0.13991611 | 0.15995341 | 0.16561478 |
| 2018-2019 | - | - | 0.16638372 | 0.16638372 |
| 2017-2018 | - | - | 0.16607318 | 0.16607318 |
| 2016-2017 | - | - | 0.16609925 | 0.16609925 |
| 2015-2016 | - | - | 0.17111581 | 0.17111581 |

†Calculated over period 1/7/2020 to 30/11/2020

Table S7 Concentration indices for Australia – entering 20 consultations for SA3s where the data has been suppressed.

| Financial Year | Video-linked | Telephone | Face-to-face | Total |
| --- | --- | --- | --- | --- |
| 2020-2021† | 0.35425500† | 0.15397445† | 0.12886598† | 0.16345855† |
| 2019-2020 | 0.32343452 | 0.13933838 | 0.15995341 | 0.16561478 |
| 2018-2019 | - | - | 0.16637668 | 0.16637668 |
| 2017-2018 | - | - | 0.16607318 | 0.16607318 |
| 2016-2017 | - | - | 0.16609206 | 0.16609206 |
| 2015-2016 | - | - | 0.17110846 | 0.17110846 |

†Calculated over period 1/7/2020 to 30/11/2020

The sensitivity analyses for the corresponding results on a state-by-state basis for rural and urban subgroups, produced results that also differed minimally, and that had no bearing on the conclusions drawn.
